# FLII and MLL1 Cooperatively Regulate Aryl Hydrocarbon Receptor-Mediated Transcription in ARPE-19 Cells

**DOI:** 10.3390/cimb43030115

**Published:** 2021-10-16

**Authors:** Kwang Won Jeong

**Affiliations:** Gachon Research Institute of Pharmaceutical Sciences, College of Pharmacy, Gachon University, 191 Hambakmoero, Yeonsu-gu, Incheon 406-799, Korea; kwjeong@gachon.ac.kr; Tel.: +82-32-820-4925

**Keywords:** aryl hydrocarbon receptor, retinal pigment epithelial cell, FLII, MLL1, BRG1, transcription

## Abstract

Aryl hydrocarbon receptors (AHRs), a class of ligand-dependent nuclear receptors that regulate cellular responses by inducing the expression of various target genes in response to external signals, are implicated in maintaining retinal tissue homeostasis. Previous studies have shown that the regulation of AHR-induced gene expression requires transcriptional co-regulators. However, it is not yet clear how chromatin remodelers, histone methyltransferases and coactivators interact during AHR-mediated gene expression in human retinal cells. In this study, we reveal that the histone methyltransferase MLL1 and the coactivator FLII are involved in AHR-mediated gene expression in retinal pigment epithelial cells. 2,3,7,8-tetrachlorodibenzo-p-dioxin (TCDD) significantly increased the expression of *CYP1A1, CYP1B1* and *AHRR* in ARPE-19 cells, whereas FLII or MLL1 depletion significantly reduced the expression of these genes induced by TCDD. Mechanistically, FLII binds to AHR in a ligand-dependent manner in ARPE-19 cells. In particular, the binding of FLII to MLL1 occurs through the GelB domain of FLII. In addition, MLL1 binds to AHR in a ligand-independent manner. FLII is involved in the recruitment of the BRG1 chromatin remodeler and MLL1 histone methyltransferase to the AHR-regulated *CYP1A1* gene region in ARPE-19 cells and consequently, plays an important role in RNA polymerase II binding and transcriptional activity by modulating chromatin accessibility. Our results identify the functions and mechanisms of action of FLII and MLL1 in AHR-induced gene expression in human retinal pigment epithelial cells.

## 1. Introduction

Age-related macular degeneration (AMD), along with glaucoma and diabetic retinopathy, is one of the three leading causes of vision loss in individuals over the age of 55 worldwide [1]. AMD is divided into dry and wet macular degeneration according to the lesion type. Eyes with the dry clinical subtype are characterized by an accumulation of extracellular lipoprotein-rich deposits under the retinal pigment epithelial (RPE) cells and within the Bruch’s membrane. These deposits include drusen, basal layer and basal linear deposits [2,3] that cause RPE dysfunction, apoptosis, activation of local inflammatory responses and ultimately retinal tissue degeneration [2,3]. In particular, RPE atrophy and regression develop into a characteristic lesion of dry AMD called geographic atrophy. Recent studies have revealed the composition of sub-RPE sediments [4,5,6]. However, the important molecular events and signaling pathways that lead to progressive RPE dysfunction and extracellular sediment generation remain unknown.

Aryl hydrocarbon receptors (AHRs) are a class of ligand-dependent nuclear receptors that regulate cellular responses by inducing the expression of various target genes in response to external signals such as xenobiotics, UV and blue wavelength light. AHRs were initially identified as receptors for environmental pollutants, such as components of cigarette smoke including polycyclic and halogenated aromatic hydrocarbons (PAH and HAH), by-products of industrial combustion, and automobile exhaust gases [7]. Subsequently, various endogenous and exogenous ligands that bind to these receptors have been identified, and these ligands are involved in a variety of physiological processes ranging from homeostasis in normal cells to the proliferation of cancer cells. Upon binding of ligands such as 2,3,7,8-tetrachlorodibenzo-p-dioxin (TCDD), the AHR dimerizes with the AHR nuclear translocator (ARNT) and activates the transcription of target genes including *CYP1A1*.

AHRs have been implicated in the maintenance of retinal tissue homeostasis [8,9]. The function of AHR in the retina, particularly in RPE cells, has attracted attention as it is known that splicing mutations in AHR are associated with retinitis pigmentosa [10]. Additionally, the downregulation of AHRs observed in conditions such as aging and disease augments carcinogen-induced retinal lesions by altering SOCS3-STAT3 signaling [11], and various AHR deficiency studies have shown that AHRs have various functions such as regulating cell matrix metabolism [11], protecting RPE cells against light or oxidative damage [8], inhibiting subretinal accumulation of microglia [12] and reducing choroidal neovascularization and collagen production. Additionally, in vivo studies suggest that AHR plays an important role in protecting RPE cells, as AHR knockout mice are characterized by accelerated age-related degeneration of RPE cells and are much more vulnerable to chronic environmental stress [8,9]. Therefore, in an effort to protect retinal cells from environmental stress, researchers have attempted to search for a safe ligand for AHR, and some positive results suggest that it has the potential to be a target molecule for AMD treatment [13,14].

Chromatin remodeling is a key step in the regulation of gene expression through nucleosome modulation in eukaryotes. BRG1, an active ATPase of the SWI/SNF chromatin remodeling complex, activates reporter gene expression in Hepa1c1c7 cells in a TCDD-dependent manner and is a ligand for AHR. Additionally, recruitment of BRG1 by AHR/ARNT to the enhancer region of the mouse *CYP1A1* gene has been confirmed in vivo, and BRG1 is required for *CYP1A1* expression [15]. In association with the chromosomal remodeling that occurs during drug induction, transient DNA transfection and chromatin immunoprecipitation (ChIP) analysis indicated the involvement of BRG1 in the enhancement of *CYP1A1* gene expression by TCDD [15]. In a previous study, we found that the AHR signaling pathway is also activated in ARPE-19 cells, although different genes are targeted compared to the gene targets in hepatocytes [16]. We also found that AHR is required for chromatin remodeling by the BRG1-containing SWI/SNF complex that regulates AHR-mediated expression of target genes in ARPE-19 cells. It has been reported that FLII binds to the SWI/SNF complex, which contains BRG1 as a core ATPase that promotes estrogen-mediated target gene expression in breast cancer cells [17]. 

These previous findings suggest that chromatin remodelers and coactivators may be closely related to each other in the regulation of AHR-induced gene expression in human retinal cells. In particular, FLII may play an important role in chromatin remodeling as it is associated with the SWI/SNF complex. Here, we investigated the relationship between FLII and the histone H3K4 methyltransferase in AHR-mediated gene expression in human retinal cells and uncovered how SWI/SNF, MLL1 and FLII are established at the AHR enhancer region.

## 2. Materials and Methods

### 2.1. Cell Culture

Human RPE cells (ARPE-19) were purchased from the American Type Culture Collection (Manassas, VA, USA). Cells were grown as a monolayer in Dulbecco’s modified Eagle’s medium F-12 (ARPE-19) obtained from Welgene (Daegu, Korea) supplemented with 10% fetal bovine serum at 37 °C and in an atmosphere containing 5% CO_2_.

### 2.2. RNA Interference

Small-interfering RNA (siRNA) experiments were performed as previously described [18]. The siRNA sequences (5′–3′) used were: siFLII(1), GCAGGUGUUUGACAACGACTTdTdT (sense) and GUCGUUGUCAAACACCUGCTTdTdT (antisense); siFLII(2), GCUGGAACACUUGUCUGUGdTdT (sense) and CACAGACAAGUGUUCCAGCTdTdT (antisense); siMLL1(1), GAUUCGAACACCCAGUUAUdTdT (sense) and AUAACUGGGUGUUCGAAUCdTdT (antisense); siMLL1(2), GCACUGUUAAACAUUCCACdTdT (sense) and GUGGAAUGUUUAACAGUGCdTdT (antisense); non-specific siRNA (siNS), UUCUCCGAACGUGUCACGUdTdT (sense) and ACGUGACACGUUCGGAGAAdTdT (antisense). siRNAs were transfected into ARPE-19 cells using Oligofectamine (Invitrogen, Carlsbad, CA, USA), according to the manufacturer’s protocol.

### 2.3. Quantitative Reverse Transcription Polymerase Chain Reaction (RT-qPCR)

Total RNA was isolated from ARPE-19 after TCDD (10 nM, 2.5 h) treatment of cells using Trizol (Invitrogen, Carlsbad, CA, USA) and reverse-transcribed using the iScript cDNA Synthesis Kit (Bio-Rad Laboratories, Hercules, CA, USA). Real-time PCR amplification of cDNA was performed using a Roche LightCycler 480 II and SYBR Green Master Mix (Roche, Indianapolis, IN, USA). The sequences of the PCR primers used in this study are listed in Table 1. Relative expression levels of the target genes were determined by normalizing to the *18S* levels. The results are depicted as means and the range of variation of duplicate PCR reactions performed on the same cDNA sample. The results shown are from a single experiment that is representative of at least two independent experiments conducted on different days.

### 2.4. Protein-Binding Assay

For co-immunoprecipitation assays, cell lysates from ARPE-19 cells treated with TCDD (10 nM) or vehicle for 24 h were prepared in 1 mL of radioimmunoprecipitation assay (RIPA) buffer (50 mM Tris-HCl (pH 8.0), 150 mM NaCl, 2 mM EDTA, 1% SDS, 1% sodium deoxycholate and 1% NP-40). Immunoblotting was performed as previously described [19] using the following antibodies: anti-FLII (sc-21716), anti-BRG1 (sc-17796), anti-AHR (sc-133088), normal mouse IgG (sc-2050, Santa Cruz Biotechnology, Dallas, TX, USA) and anti-MLL1 (AP6182a, Abcepta, San Diego, CA, USA). For in vitro binding assays, GST-fused LRR, GelA or GelB domains of FLII were expressed in *E. coli* and incubated with ARPE-19 cell lysates. After pulldown using glutathione beads, immunoblotting was performed using anti-MLL1, anti-ASH2L (sc-21716, Santa Cruz Biotechnology, Dallas, TX, USA) or anti-RBBP5 (#13171, Cell Signaling Technology, Danvers, MA USA) antibodies.

### 2.5. Chromatin Immunoprecipitation (ChIP) Assay

ChIP assays were performed according to previously described protocols [20]. ARPE-19 cells were treated with TCDD (10 nM) or vehicle for 20 min. Immunoprecipitation was conducted with anti-AHR, anti-FLII, anti-MLL1 (A300-086A, Bethyl, Montgomery, TX, USA) and anti-BRG1 (A2024050, Epigentek, Farmingdale, NY, USA) antibodies. The sequences of the primers used in ChIP-qPCR are listed in Table 1. The results are expressed as the percentage of input chromatin (before immunoprecipitation).

### 2.6. Formaldehyde-Assisted Isolation of Regulatory Elements (FAIRE)-qPCR

FAIRE-qPCR was performed as previously described [21]. Cells were treated with 10 nM TCDD or vehicle for 1 h. Results are presented as means and the range of variation of duplicate PCR reactions performed on the same DNA sample. Results are expressed as the percentage of input chromatin (Input) and were derived from a single experiment that is representative of at least two independent experiments. Sequences of the FAIRE-qPCR primers are the same as those used for ChIP-qPCR analysis (Table 1).

### 2.7. Statistical Analysis

The results of RT-qPCR and ChIP-qPCR were statistically analyzed by Student’s *t*-test (two-tailed) using Prism 5.0 (Graphad Software, San Diego, CA, USA). Data are presented as means ± standard deviation (S.D., n = 3). Results with *P* < 0.05 were considered statistically significant. (* *P* < 0.05).

## 3. Results

### 3.1. FLII and MLL1 Are Involved in AHR-Mediated Transcription in ARPE-19 Cells

**Figure 1:**
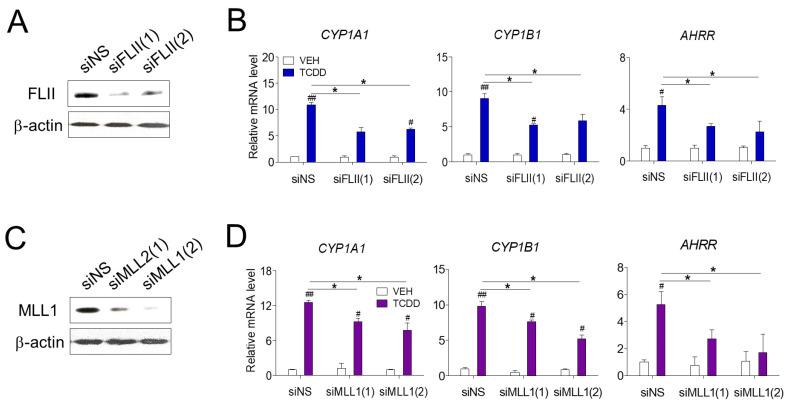
AHR-mediated transcription in ARPE-19 cells. (**A**,**B**) Reduction in the levels of endogenous FLII and MLL1 by siRNA. ARPE-19 cells were transiently transfected with siRNAs targeting two different sites of FLII mRNA (siFLII(1) or siFLII(2)), MLL1 (siMLL1(1) or siMLL1(2)), or non-specific siRNA (siNS) for 48 h. Endogenous FLII and MLL1 protein levels in ARPE-19 cells were measured by western blotting. (**C**,**D**) Reduction in the levels of endogenous FLII and MLL1 by siRNA attenuated the expression of AHR target genes. The expression of AHR target genes in ARPE-19 cells was analyzed by RT-qPCR. Cells were treated with 10 nM TCDD or vehicle (toluene). Data are presented as means ± S.D. (n = 3). * *P* < 0.05 vs. siNS, # *P* < 0.05 and ## *P* < 0.01 vs. vehicle control.

We first investigated the role of FLII in AHR-mediated gene expression in RPE cells. Transfection of siFLII(1) or siFLII(2) targeting two different sites of FLII mRNA effectively reduced the mRNA and protein levels of endogenous FLII in ARPE-19 cells (Figure 1A). TCDD treatment significantly increased the expression of known AHR target genes (*CYP1A1, CYP1B1* and *AHRR*) in ARPE-19 cells (Figure 1B). In contrast, siFLII transfection significantly decreased the expression of TCDD-induced *CYP1A1, CYP1B1* and *AHRR* compared to the levels in cells transfected with non-specific siRNA (siNS). Next, we investigated the effect of the MLL1 histone methyltransferase on AHR-mediated gene expression. Cells transfected with siMLL1 showed a significant decrease in the expression of *CYP1A1, CYP1B1* and *AHRR* induced by TCDD compared to the levels in cells transfected with non-specific siRNA (Figure 1C,D). It is known that other coactivators, such as p160, p300/CBP, PRMTs (protein arginine methyltransferases) and CARM1 (coactivator associated arginine methyltransferase 1), act together in the AHR-mediated regulation of gene expression by TCDD [22]. Therefore, despite the effective knockdown of FLII or MLL1, a degree of expression of *CYP1A1* is presumably due to the regulatory action of other coactivators. These results suggest that MLL1, a histone methyltransferase, and FLII, a coactivator, are involved in AHR-mediated gene expression in RPE cells.

### 3.2. FLII Interacts with AHR and MLL1 in ARPE-19 Cells

**Figure 2:**
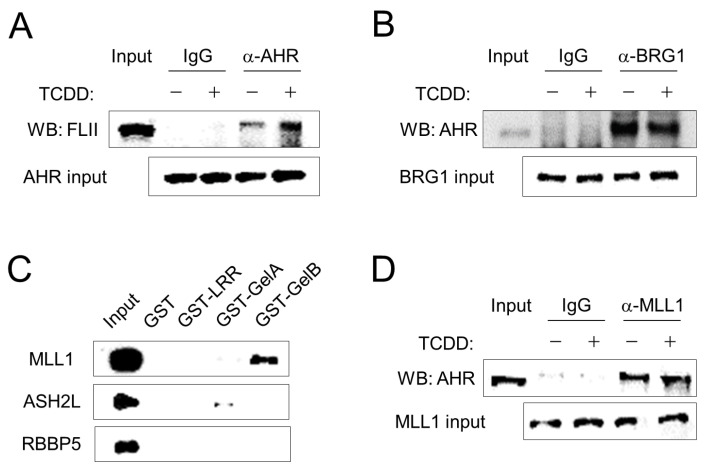
FLII interacts with AHR and MLL1 in ARPE-19 cells. (**A**) Co-immunoprecipitation was performed with total extracts from ARPE-19 cells. Cells were treated with either 10 nM TCDD or vehicle (toluene). One hour later, cells were harvested and total cellular protein was extracted with RIPA buffer. Immunoprecipitation was performed using normal IgG or an anti-AHR antibody. Immunoblotting was performed using an anti-FLII antibody. (**B**) AHR binding to BRG1. Immunoprecipitation was performed using normal IgG or an anti-BRG1 antibody. Immunoblotting was performed using an anti-AHR antibody. (**C**) In vitro protein-binding assay. GST-fused LRR, GelA or GelB domains of FLII expressed in *E. coli* were immobilized on glutathione agarose beads and then incubated with ARPE-19 cell lysates for 24 h. Bound MLL1, ASH2L or RBBP5 were analyzed by western blotting using specific antibodies. (**D**) AHR binding to MLL1. Immunoprecipitation was performed using normal IgG or an anti-MLL1 antibody. Immunoblotting was performed using an anti-AHR antibody.

To elucidate the mechanism of AHR target gene regulation by FLII and MLL1, the interaction between these proteins in ARPE-19 cells was examined by co-immunoprecipitation assay. FLII was associated with AHR, and their binding was further promoted by treatment with TCDD, a ligand of AHR (Figure 2A). BRG1, the core ATPase of the SWI/SNF complex, was also associated with AHR, and its interaction was not affected by the ligand (Figure 2B). Next, we investigated the binding between FLII and MLL1. FLII is structurally composed of a leucine-rich repeat (LRR) domain at the N-terminus and a gelsolin-like domain at the C-terminus. In addition, the gelsolin-like domain is composed of six Gel subunits and is functionally divided into a GelA domain including three Gel subunits at the N-terminus and a GelB domain including 3 Gel subunits at the C-terminus. We used a recombinant protein in which these three FLII domains (LRR, GelA and GelB) were fused to GST. Of the three domains, the GelB domain was most strongly bound to MLL1 (Figure 2C). In contrast, ASH2L and RBBP5, the partner proteins that interact with MLL1 [23], did not bind to FLII. Finally, we investigated the binding between AHR and MLL1. MLL1 was bound to AHR in a ligand-independent manner (Figure 2D). Overall, FLII and MLL1 are associated with AHR in ARPE-19 cells through individual binding to AHR as well as mutual binding between FLII and MLL1 via GelB. In addition, the binding of FLII to the SWI/SNF complex [17] and the binding of AHR to BRG1 (Figure 2B) suggest the possibility of the functional involvement of these proteins in the transcription process.

### 3.3. FLII and MLL1 Are Recruited to the CYP1A1 in ARPE-19 Cells

**Figure 3:**
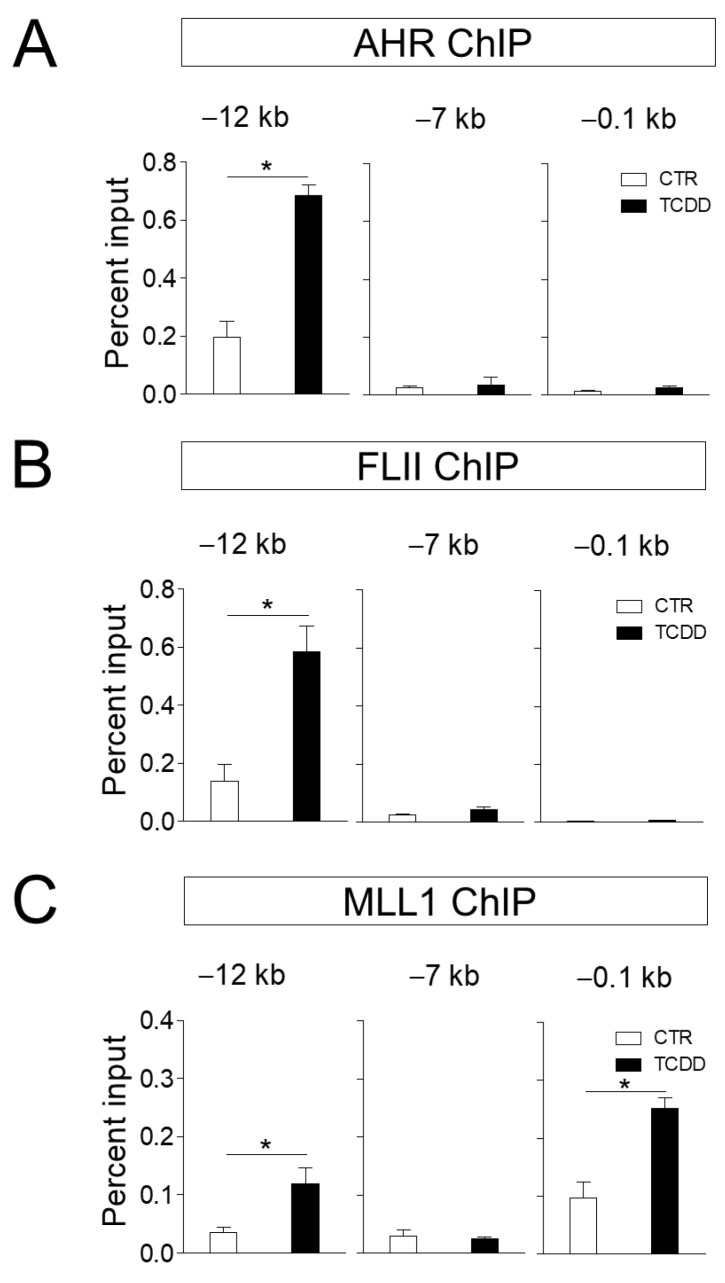
FLII and MLL1 are recruited to the *CYP1A1* enhancer. (**A–C**) Chromatin immunoprecipitation assays were performed with ARPE-19 cells treated with 10 nM TCDD or vehicle for 20 min in 150-mm dishes. After immunoprecipitation of cross-linked chromatin fragments with the AHR, FLII or MLL1 antibody, precipitated DNA was analyzed by qPCR with primer sets of −12 (enhancer), −0.1 (promoter) and −7 kb (control) from the transcription start site of the *CYP1A1* gene. Data are presented as means ± S.D. (n = 3). * *P* < 0.05 vs. control.

Next, we investigated whether FLII and MLL1 regulate gene expression at the transcriptional level at the target gene site of AHR. Our ChIP results confirmed that AHR was recruited in a ligand-dependent manner to the −12 kb enhancer site of the *CYP1A1* gene. We previously identified the −12 kb region of the *CYP1A1* gene to be an enhancer in ARPE-19 cells and confirmed that chromatin remodelers such as BRG1 and AHR [16]. are co-recruited to this site (Figure 3A). Additional ChIP assays revealed that FLII was also bound to this enhancer site in a ligand-dependent manner (Figure 3B). On the other hand, MLL1 was recruited to the promoter and enhancer sites, and its binding was ligand dependent (Figure 3C). MLL1 catalyzes the trimethylation of histone H3K4 residues, and H3K4me3 is one of the key features of the promoter region of active genes. Following trimethylation of H3K4 by the SET domain of MLL1, the PHD domain of MLL1 binds to H3K4me3 with high affinity and specificity, helping localization of MLL1 to the promoter region [23].

### 3.4. FLII Is Required for the Chromatin Structure at the CYP1A1 Locus in ARPE-19 Cells

**Figure 4:**
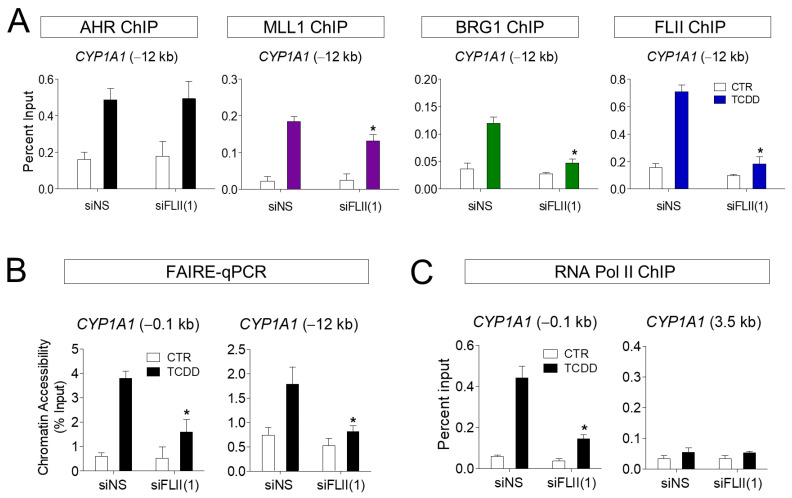
FLII is required for the chromatin structure at the *CYP1A1* locus in ARPE-19 cells. (**A**) Chromatin immunoprecipitation assays in FLII-depleted ARPE-19 cells. ARPE-19 cells were transiently transfected with siFLII(1) or siNS for 48 h. The amount of the precipitated *CYP1A1* enhancer fragments was determined by qPCR. (**B**) FLII is required for chromatin accessibility at the *CYP1A1* enhancer region. Chromatin accessibility at the *CYP1A1* enhancer was assessed by FAIRE-qPCR analysis using chromatin samples prepared from ARPE-19 cells transfected with siNS or siFLII(1) and treated with 10 nM TCDD or vehicle for 1 h. (**C**) Chromatin immunoprecipitation assay using an RNA polymerase II antibody was performed as in Figure 4A. 3.5 kb region of *CYP1A1* was used as control. Data are presented as means ± S.D. (n = 3). * *P* < 0.05 vs. control.

Our previous results suggest that the recruitment of AHR to the target gene site, as well as that of FLII and MLL1, was increased by ligand treatment. To understand the function of FLII in this process, the binding of FLII and MLL1 to the *CYP1A1* locus (−12 kb) was investigated in ARPE-19 cells with FLII knocked down. First, knockdown (KD) of FLII had no effect on the recruitment of AHR to the *CYP1A1* enhancer site. In contrast, recruitment of MLL1 and BRG1 induced by TCDD was significantly reduced by FLII KD (Figure 4A). In addition, we confirmed by FAIRE-qPCR assays that FLII KD significantly reduced chromatin accessibility in the promoter and enhancer regions of *CYP1A1* in ARPE-19 cells (Figure 4B). Consistent with this, FLII KD significantly reduced the binding of RNA polymerase II to the promoter region of *CYP1A1* (Figure 4C). It is noteworthy that MLL1 and BRG1 can bind directly to AHR as well as FLII. Owing to the binding of MLL1 and BRG1 to AHR, it is thought that recruitment of these proteins to the CYP1A1 locus remains, to some extent, even after FLII knockdown. Therefore, the knockdown of FLII alone is insufficient to completely block recruitment of MLL1 and BRG1 to the CYCP1A1 gene. However, FLII is apparently required for the stable formation of these protein complexes, and for optimal AHR-mediated transcription. Taken together, these observations indicate FLII is involved in the recruitment of BRG1 and MLL1 to the AHR-regulated *CYP1A1* locus in ARPE-19 cells upon ligand treatment, and consequently plays an important role in transcription through chromatin accessibility.

## 4. Discussion

AHR is a nuclear receptor that regulates the expression of genes responsible for the metabolism and detoxification of xenobiotic. AHR activity in the retina decreases with aging, and this process has been linked to dry AMD [9]. The function of AHR in the retina was confirmed using a mouse knockout model. AhR-/- mice show loss of RPE cell tight junctions; accumulation of RPE cell lipofuscin; basal laminar, linear-like deposit material; Bruch’s membrane thickening; progressive RPE and choroidal atrophy; and choroidal neovascularization [8,9,24] Consistent with this, treatment with the AHR agonist significantly lowered diabetes-mediated leukocyte retention, oxidative stress and inflammation in streptozotocin-induced retinopathy, demonstrating that AHR could potentially represent a novel therapeutic target for diabetic retinopathy [14].

Previous studies have shown that the promotion of target gene expression by AHR requires BRG1 in Hepa1c1c7 cells [15]. Furthermore, we recently demonstrated that the AHR signaling pathway is activated in ARPE-19 cells, and that BRG1 directly interacts with the C-terminal activation domain of AHR to regulate target gene expression [16]. BRM and BRG1 mediate chromatin remodeling by binding to AHR target loci and recruiting steroid receptor coactivator 1 (SRC-1), steroid receptor coactivator 2 (SRC-2) and p300, a nuclear receptor coactivator in breast cancer cells [25]. We also found that AHR is required for chromatin remodeling by the BRG1-containing SWI/SNF complex that regulates the AHR-mediated expression of target genes in ARPE-19 cells [25].

In the present study, we showed that the histone H3K4 methyltransferase MLL1 and the coactivator FLII are involved in AHR-mediated gene expression in RPE cells. The expression of AHR target genes such as *CYP1A1, CYP1B1* and *AHRR* that is induced by TCDD in ARPE-19 cells was significantly reduced by FLII depletion. FLII can directly bind to BAF53, an actin-related component of the SWI/SNF complex, and this interaction is essential for SWI/SNF to bind its target genes and remodel chromatin [17]. Consequently, FLII serves as a key transcriptional regulator by modulating the accessibility of RNA polymerase II and the coactivators on chromatin [26]. Chromatin remodeling by the SWI/SNF complex induces the binding of histone modifiers such as histone methyltransferases. Previous studies have shown that FLII regulates SENP3 recruitment and MLL1/2 complex assembly at the DLX3 locus in human mesenchymal stem cells [27]. These results suggest that FLII is required for H3K4 methylation and the proper binding of RNA polymerase II to the target locus [27]. We also demonstrated that FLII binds to MLL1 and AHR in a ligand-independent manner in ARPE-19 cells via its GelB domain and is involved in the recruitment of the BRG1-chromatin remodeler and MLL1 histone methyltransferase to the *CYP1A1* gene site. Consequently, chromatin accessibility has been shown to play an important role in RNA polymerase II binding and transcriptional activity. Taken together, our results uncovered that chromatin-remodelers, histone methyltransferases and coactivators are established at the enhancers and promoters of AHR target genes in RPE cells to regulate transcription. The growing understanding of the function of AHR in various diseases, in addition to ophthalmic diseases, is fueling attempts to target AHR in the treatment of diseases, including AMD, certain tumors, immune disorders and inflammatory diseases. Advances in AHR-mediated transcriptional regulation identified through our study are expected to inspire the development of tissue-specific AHR agonists or antagonists in the future.

## Figures and Tables

**Table 1:** Primer sequences for qPCR.

Name	Assay Type	Forward	Reverse
*18S*	RT-qPCR	GAGGATGAGGTGGAACGTGT	TCTTCAGTCGCTCCAGGTCT
*CYP1A1*	RT-qPCR	TGAACCCCAGGGTACAGAGA	GGCCTCCATATAGGGCAGAT
*CYP1B1*	RT-qPCR	AACGTACCGGCCACTATCAC	CCACGACCTGATCCAATTCT
*AHRR*	RT-qPCR	AGACTCCAGGACCCACAAAG	CATCCTCACTGTGCTTTCCC
*CYP1A1* (−12 kb)	ChIP	AGTGGCTCACGCCAGTAATC	CGTGTTAGCCAGGATGGTCT
*CYP1A1* (−7 kb)	ChIP	GTAGAGACGGGGTTTCACCA	GTGGCTCACGCCTATAATCC
*CYP1A1* (−0.1 kb)	ChIP	CGTACAAGCCCGCCTATAAA	CTGGGATCACAAGGATCAGG
*CYP1A1* (3.5 kb)	ChIP	CATGTCGGCCACGGAGTTTCTTC	ACAGTGCCAGGTGCGGGTTCTTTC

## Data Availability

The raw data used and/or analyzed during the current study will be available from the corresponding author on reasonable request.

## References

[1] Klein R., Lee K.E., Gangnon R.E., Klein B.E. (2013). Incidence of visual impairment over a 20-year period: The Beaver Dam Eye Study. Ophthalmology.

[2] Sarks S., Cherepanoff S., Killingsworth M., Sarks J. (2007). Relationship of Basal laminar deposit and membranous debris to the clinical presentation of early age-related macular degeneration. Investig. Ophthalmol. Vis. Sci..

[3] Curcio C.A., Millican C.L. (1999). Basal linear deposit and large drusen are specific for early age-related maculopathy. Arch. Ophthalmol..

[4] Anderson D.H., Radeke M.J., Gallo N.B., Chapin E.A., Johnson P.T., Curletti C.R., Hancox L.S., Hu J., Ebright J.N., Malek G. (2010). The pivotal role of the complement system in aging and age-related macular degeneration: Hypothesis re-visited. Prog. Retin. Eye Res..

[5] Crabb J.W., Miyagi M., Gu X., Shadrach K., West K.A., Sakaguchi H., Kamei M., Hasan A., Yan L., Rayborn M.E. (2002). Drusen proteome analysis: An approach to the etiology of age-related macular degeneration. Proc. Natl. Acad. Sci. USA.

[6] Wang L., Clark M.E., Crossman D.K., Kojima K., Messinger J.D., Mobley J.A., Curcio C.A. (2010). Abundant lipid and protein components of drusen. PLoS ONE.

[7] Phillips D.H. (1999). Polycyclic aromatic hydrocarbons in the diet. Mutat. Res..

[8] Kim S.Y., Yang H.J., Chang Y.S., Kim J.W., Brooks M., Chew E.Y., Wong W.T., Fariss R.N., Rachel R.A., Cogliati T. (2014). Deletion of aryl hydrocarbon receptor AHR in mice leads to subretinal accumulation of microglia and RPE atrophy. Investig. Ophthalmol. Vis. Sci..

[9] Hu P., Herrmann R., Bednar A., Saloupis P., Dwyer M.A., Yang P., Qi X., Thomas R.S., Jaffe G.J., Boulton M.E. (2013). Aryl hydrocarbon receptor deficiency causes dysregulated cellular matrix metabolism and age-related macular degeneration-like pathology. Proc. Natl. Acad. Sci. USA.

[10] Zhou Y., Li S., Huang L., Yang Y., Zhang L., Yang M., Liu W., Ramasamy K., Jiang Z., Sundaresan P. (2018). A splicing mutation in aryl hydrocarbon receptor associated with retinitis pigmentosa. Hum. Mol. Genet..

[11] Tsai C.H., Lee Y., Li C.H., Cheng Y.W., Kang J.J. (2020). Down-regulation of aryl hydrocarbon receptor intensifies carcinogen-induced retinal lesion via SOCS3-STAT3 signaling. Cell Biol. Toxicol..

[12] Esfandiary H., Chakravarthy U., Patterson C., Young I., Hughes A.E. (2005). Association study of detoxification genes in age related macular degeneration. Br. J. Ophthalmol..

[13] Gutierrez M.A., Davis S.S., Rosko A., Nguyen S.M., Mitchell K.P., Mateen S., Neves J., Garcia T.Y., Mooney S., Perdew G.H. (2016). A novel AhR ligand, 2AI, protects the retina from environmental stress. Sci. Rep..

[14] Zapadka T.E., Lindstrom S.I., Batoki J.C., Lee C.A., Taylor B.E., Howell S.J., Taylor P.R. (2021). Aryl Hydrocarbon Receptor Agonist VAF347 Impedes Retinal Pathogenesis in Diabetic Mice. Int. J. Mol. Sci..

[15] Wang S., Hankinson O. (2002). Functional involvement of the Brahma/SWI2-related gene 1 protein in cytochrome P4501A1 transcription mediated by the aryl hydrocarbon receptor complex. J. Biol. Chem..

[16] Jin H.L., Jeong K.W. (2016). Regulation of aryl hydrocarbon receptor-mediated transcription in human retinal pigmented epithelial cells. Biochem. Biophys. Res. Commun..

[17] Jeong K.W., Lee Y.H., Stallcup M.R. (2009). Recruitment of the SWI/SNF chromatin remodeling complex to steroid hormone-regulated promoters by nuclear receptor coactivator flightless-I. J. Biol. Chem..

[18] Chung Y.S., Jin H.L., Jeong K.W. (2020). Cell-specific expression of ENACalpha gene by FOXA1 in the glucocorticoid receptor pathway. Int. J. Immunopathol. Pharmacol..

[19] Yang L., Jin M., Park S.J., Seo S.Y., Jeong K.W. (2020). SETD1A Promotes Proliferation of Castration-Resistant Prostate Cancer Cells via FOXM1 Transcription. Cancers.

[20] Yang L., Jin M., Jung N., Jeong K.W. (2020). MLL2 regulates glucocorticoid receptor-mediated transcription of ENACalpha in human retinal pigment epithelial cells. Biochem. Biophys. Res. Commun..

[21] Jin H.L., Choi Y., Jeong K.W. (2017). Crosstalk between Aryl Hydrocarbon Receptor and Glucocorticoid Receptor in Human Retinal Pigment Epithelial Cells. Int. J. Endocrinol..

[22] Endler A., Chen L., Shibasaki F. (2014). Coactivator recruitment of AhR/ARNT1. Int. J. Mol. Sci..

[23] Yang L., Jin M., Jeong K.W. (2021). Histone H3K4 Methyltransferases as Targets for Drug-Resistant Cancers. Biology.

[24] Choudhary M., Kazmin D., Hu P., Thomas R.S., McDonnell D.P., Malek G. (2015). Aryl hydrocarbon receptor knock-out exacerbates choroidal neovascularization via multiple pathogenic pathways. J. Pathol..

[25] Taylor R.T., Wang F., Hsu E.L., Hankinson O. (2009). Roles of coactivator proteins in dioxin induction of CYP1A1 and CYP1B1 in human breast cancer cells. Toxicol. Sci. Off. J. Soc. Toxicol..

[26] Lim M.S., Jeong K.W. (2014). Role of Flightless-I (Drosophila) homolog in the transcription activation of type I collagen gene mediated by transforming growth factor beta. Biochem. Biophys. Res. Commun..

[27] Nayak A., Reck A., Morsczeck C., Muller S. (2017). Flightless-I governs cell fate by recruiting the SUMO isopeptidase SENP3 to distinct HOX genes. Epigenetics Chromatin.

